# Group decision-making in chacma baboons: leadership, order and communication during movement

**DOI:** 10.1186/1472-6785-11-26

**Published:** 2011-10-20

**Authors:** Cédric Sueur

**Affiliations:** 1 Department of Ecology and Evolutionary Biology, Princeton University, Princeton, USA; 2 Centre National de la Recherche Scientifique, Département Ecologie, Physiologie et Ethologie, Strasbourg, France; 3 Université de Strasbourg, Institut Pluridisciplinaire Hubert Curien, Strasbourg, France; 4 Unit of Social Ecology, free University of Brussels, Brussels, Belgium

## Abstract

**Background:**

Group coordination is one of the greatest challenges facing animals living in groups. Obligatory trade-offs faced by group members can potentially lead to phenomena at the group level such as the emergence of a leader, consistent structure in the organization of individuals when moving, and the use of visual or acoustic communication. This paper describes the study of collective decision-making at the time of departure (i.e. initiation) for movements of two groups of wild chacma baboons (*Papio ursinus*). One group was composed of 11 individuals, whilst the other consisted of about 100 individuals.

**Results:**

Results for both groups showed that adult males initiated more movements even if the leadership was also distributed to adult females and young individuals. Baboons then joined a movement according to a specific order: adult males and adult females were at the front and the back of the group, sub-adults were at the back and juveniles were located in the central part of the progression. In the two groups, vocalisations, especially loud calls, were more frequently emitted just before the initiation of a group movement, but the frequency of these vocalisations did not influence the success of an initiation in any way.

**Conclusion:**

The emergence of a leadership biased towards male group members might be related to their dominance rank and to the fact that they have the highest nutrient requirements in the group. Loud calls are probably not used as recruitment signals but more as a cue concerning the motivation to move, therefore enhancing coordination between group members.

## Background

Animals living in groups have to synchronise their activities and coordinate their movements in order to remain cohesive [1-4]. Group members therefore have to reach consensus about the time and the direction to move collectively [5]. However, animals in heterogeneous groups differ in their nutrient requirements, in the information they have about the environment or in their ability to monopolise resources. These differences lead to conflicts of interest between group members, which could result in different strategies emerging about the best way to collectively decide where and when to move [6]. These strategies could be summarised and observed via three different explorations: 1) who leads the groups and at what frequency, 2) how individuals are organized during a group movement and 3) what form of communication, if any, is used at the start of group movements, or during them.

The term "leadership" commonly refers to individuals initiating movements or to individuals who change the direction of movement and are followed by the rest of the group [7-10]. Even if more and more studies show that this leadership is distributed among all group members, some individuals are seen to initiate movements more often, or are more often located at the front of the progression than their conspecifics [7,8,10-12]. Several theories have been proposed to explain this emergence of leadership, and they can be separated into two models: the conflict model and the integrative (or voluntary) model [13]. In the first model, the leader forces its conspecifics to follow him. In the second model, the emergence of a leader within the group "may in some cases be preferred over egalitarian arrangements [...] as a solution to the key challenges of life in social groups, such as conflicts over resources, coordination failures and free-riding in cooperative relationships" according to Hooper and collaborators [13]. In this last model, the leader does not impose a decision on its conspecifics: the latter "decide to let one individual decide". Several individuals or social factors influence the leadership [3,6]. Old individuals are presumed to have a better knowledge of the environment and may therefore lead the group to rare food resources. Many instances have been described in which aged individuals led the group more often than other group members (for instance, see rhesus macaques, *Macaca mulatta*: [14]). The most striking example however is most probably the matriarch in elephants [15]. Dominance is also a factor affecting leadership. In the mountain gorilla (*Gorilla gorilla berengei*), the silverback walks quickly in the direction of the future movement and the other group members follow him [16]. There is however an increase in frequency of grunts emitted by females before the initiation of the silverback [17]. Similar initiations by dominants have also been described in wolves (*Canis lupus*, [18]) and in mongooses (*Helogale parvula*, [19]). However, high-ranking individuals are often those with the highest body mass, and thus the highest nutrient requirements. The different needs of individuals also have an impact on initiation frequency. Lactating or pregnant females decide more often than the other group members in baboons [20] or in zebras (*Equus burchellii*, [21]). A study on the emergence of leadership based on nutrient requirements showed that the individual having the highest needs becomes the leader in about 80% of collective movements. This decision-making system is viable: all individuals are satisfied and can also meet their energetic needs throughout the day, even if they rarely make the decision for the group to move [22]. For instance, the body mass of adult chacma baboon males is around 29 kg whilst females weigh between 16 and 20 kg [23,24]. However, lactating females need about 200% more protein and water than non-lactating females [25].

Following is possibly as important as leading [2,26]. Certain advantages could be gained from the rank held by individuals within the progression (in this article, the term "rank" indicates the position of an individual within the progression order, and is not related to dominance hierarchy in any way). For instance, dominant males or lactating females are at the front of the progression, where they have better access to food resources and can decide which direction to take (African buffalos, *Syncerus *caffer, [27]; zebras, [28]; yellow baboons, *Papio cynocephalus*, [29]; chimpanzees, *Pan troglodytes*, [30]). On the other hand, juveniles or some females could also be in the central part of the progression simply because these ranks offer the best protection against predators [31-33]. Some authors suggest that this type of order within the progression of primates should be attributed to their high cognitive abilities [34,35]: " In a species as intelligent as the baboon, almost any form of social behaviour, spatial or otherwise, could understandably exhibit considerable variation arising from the baboon's ability to adapt features of his world" [29]. However, recent research in primates shows that the complex patterns we observed during collective movements can be explained by self-organised processes that have also been described in species such as insects and ungulates [36-39]. There is no real necessity for each group member to know its conspecifics individually or to have a global view of the collective phenomenon [4,36,40]: an estimation of the number of individuals already moving and/or the number of individuals still resting is sufficient to produce complex collective phenomena. These "rules of thumb" are based on physiological needs and social relationships [4,40], and they have explained departure latencies, order and associations of individuals during movements in macaques [41] and lemurs [42]. Whilst former studies tend to prove that the whole group decides to move only through the initiator's act (leadership hypothesis, see [42] for details), an increasing number of recent studies now show that following behaviour depends on mimetism (or social facilitation [43] i.e., the probability of an individual to display a behaviour depends on the number of individuals already performing this behaviour [4,40]) and that the initiator is not the only decision-maker for a group movement [37,38,41,44-48].

Collective decision-making cannot be achieved if group members do not exchange information about their state or their motivations. This communication does not need to be complex and can simply rely on local interactions [6,9,37]. The most basic signal is walking in a specific direction, showing that the individual is now motivated to go in that particular direction [49]. Even if vocalisations are used, studies on macaques [10,50] and chacma baboons [9] showed that this simple visual signal is enough to initiate a movement and to make all group members join it. The same conclusion is reached in the case of non primate species such as geese (*Anser domesticus*, [51]), cattle (*Bos Taurus*, [52]) and sheep (*Ovis aries*, [53]). Authors have however observed that acoustic signals, such as a 'coo' vocalization in Japanese macaques (*Macaca fuscata*, [54]) and 'trill' in the white-faced capuchin monkey (*Cebus capucinus*, [34]) are also given before the departure of groups. The acoustic signal is maybe more advantageous in primates than a visual signal, as it has the advantage of propagating the information over a longer distance without being affected by physical barriers [55]. Authors reported that in baboons, vocalisations such as grunts or loud calls ('wahoos') may play a role in the cohesion of group members [56-60]. Although all individuals emit grunts, loud calls are only emitted by males and depend on the dominance rank with high-ranking individuals showing a greater probability to emit grunts [59,60]. However, these signals are not displayed or do not have influence on collective movements in some groups of baboons [9].

In this study, I attempt to identify collective decision-making mechanisms in two groups of free-ranging chacma baboons, namely one small group of 11 individuals and one large group of about 100 individuals. First, the emergence of leadership, organization of individuals and communication had never been studied at the same time on a same group. Secondly, no previous study has assessed how group size might affect these three variables during group movements. I assess in turn the distribution of leadership in both groups, the organization of group members after departure, and communication used by individuals to reach a consensus. I expect adult males to be the main leaders of the group, mainly because of their higher nutrient requirements or their dominance rank [22,61]. For progression order, adult males and sub-adults should rather be located at the front and the back of the progression (the distribution of ranks should follow a parabolic curve, for instance) whilst adult females and juveniles should be found more in the middle of the progression (the distribution of ranks should follow an inverse parabolic curve) for a better protection against predators. If there is a possibility of vocalizations improving coordination between members, I suggest that vocalizations, and particularly loud calls, should be particularly emitted before the initiation of a movement and in the large group. The results of the two groups will be compared in order to assess whether or not the mechanisms underlying decision-making differ according to group size.

## Methods

### Study site and subjects

Data on chacma baboons were scored at the Wildcliff Nature Reserve, Western Cape, South Africa (33.959997°N, 21.034478°E) from May to July 2009 for the large group and from May to July 2010 for the small group. The reserve is a mountain wilderness reserve consisting of deep ravines with afro-mountain forest, rocky mountain tops and high fynbos meadows. An invasive plant, the black wattle, and a grassy meadow can also be found on the reserve. Direct or indirect cues showed that leopards and other small carnivores were present in the reserve. Three groups of chacma baboons populate this reserve and its surroundings: a large group, a small group and a third group entirely composed of males. At the time of the study, the large group consisted of between 95 and 105 individuals (about 9.1% were adult males, 37% were adult females without babies (< 1 year), 5.6% were adult females with babies, 16.5% were sub-adults (4-6 years old) and 31.8% were juveniles (1-3 years old)). The small group consisted of 11 individuals: one adult male, five adult females (without babies), two sub-adults and four juveniles. The compositions of these two groups are therefore comparable. The two groups were already habituated to the presence of human beings. Group members could not be identified individually, only classes of sex and age were identifiable (see definitions of classes below). A previous study showed that movement of these baboons throughout the day are relative to foraging and finding resources [62].

### Definitions

An 'initiation' was defined as the movement of an individual in one direction over a distance of at least 20 m beyond the periphery of the group, when no other initiation had been made in the same direction within the 15 previous minutes (as used in previous studies on primates: [7,9,10,26,63]). An individual performing this behaviour is called the 'initiator'. All departing movements in the same direction as the initiator within the 15 minutes following the initiation were considered to be following movement, and the individuals performing these behaviours for distances of 20 meters or more were defined as followers (as defined in previous studies [7,9,10,26,63]). If no group members followed the initiator within 15 minutes, the phenomenon was defined as a 'failed initiation'. I scored the number of followers for each initiation and analysed it. I did not define a threshold to qualify an initiation as 'successful'. Indeed, it is difficult to predict exactly how many followers are necessary to make an initiation successful for an initiator, even if previous research has revealed a threshold of three followers in capuchins [45] and macaques [50] when an initiator stops to emit recruitment signals. In baboons, Stueckle and Zinner [9] set the threshold at five following individuals to qualify an initiation as successful.

The order of progression during departure (lag between the initiation and the adhesion of the last follower) was calculated. The initiator was ranked 1, the first follower was ranked 2 and the rank of the j^th ^follower was ranked j+1. When two individuals joined the movement at the same time, I considered the two individuals to have an identical rank. The joining time for all participants is defined as the time between the departure of the initiator and the departure of the last follower. The departure latency of a follower *j *is the time between the departure of follower *j *and the departure of the previous follower *j - 1*.

### Data collection

Accompanied by a field assistant, I scored the location of the baboon group from dawn (about 7:00) to dusk (about 17:00) throughout the study period.

Initiations were only retained if at least 75% of individuals were visible and no agonistic interaction was observed [9,10,26]. 64 movements were recorded over 23 observation days for the large group, compared to 61 movements over 25 observation days in the small group. Morning departures account for 14 movements in the large group and 10 in the small group respectively. Each movement is considered as an independent event (see definition above for the time between two movements) since it is defined as a combination of several variables that are different from one movement to another [7,9,10,26,63,64].

I split individuals into five categories: adult males, adult females without infant, adult females with infant, sub-adults and juveniles. I recorded the behaviour of individuals simultaneously because the focal individual sampling method is not appropriate for studying collective decisions [9,10,49]. Data were collected using Cyber Tracker 3.0 (Cyber Tracker Conservation, Bellville, SA) with a PDA Asus 620 and a Palm Treo 750. During movement departures, I scored the rank, the time of departure and the individual category (see above) of each individual joining a movement. The field assistant used continuous sampling to score the vocalisations associated with movements (loud calls and grunts) both during movement and outside the moving context. Only the frequencies of vocalisations at the group level were analysed in this study. It was often impossible to identify the emitters of vocalisations within the larger group.

### Data analyses

Leadership: I compared the absolute frequency of initiations (i.e., number of initiations per category) between categories and between groups using a Chi square test. The corrected number of initiations was defined as the number of initiations observed per category divided by the ratio of individuals of this category within the group (for instance, 9.1% of adult males for the large group, see above). This corrected number of initiations per category was then divided by the sum of all corrected numbers of initiations for all categories in order to obtain a relative frequency of initiations or relative leadership. The shape of the relative leadership distribution was analysed using a curve estimation test (curve estimation regression statistics) in order to measure how this leadership varies between categories. The ranking of categories from the highest to the lowest leadership rate made it possible to test the shape of the distribution; see [46,65] for similar analyses. The relative frequency per category was then compared to the mean body mass of each category using a curve estimation test. Animals were not weighted, meaning that body mass was estimated on the basis of the mean documented mass for each general age-sex category [23,24]. Even if the measure is not exact, it provides information about the relation between the leadership rate and the body mass. A comparison was carried out between the different categories for the number of followers and the joining time for all participants via Mann-Whitney testing in the small group and using a Kruskal-Wallis test in the large group. I also analysed the distribution (absolute frequency and cumulative relative frequency) of the number of followers for each group using curve estimation tests. Distribution analysis is useful here to assess whether the group is either cohesive or clustered in different sub-groups.

Joining and order during progression: A survival analysis was carried out on departure latencies; the shape of the inverse cumulative distribution of departure latencies was analysed using curve estimation tests. A shape following an exponential law means that the probability of individuals joining the movement is not dependent on time, but rather on the number of individuals that already joined the movement, whilst a shape following a linear curve means that the probability is time-dependent. The shape of the mean departure latencies distribution per rank was analysed using the same test. The joining process is suggested to be mimetic if this distribution is parabolic [40]. I calculated the mean order of progression for each category of individuals to be the frequency at which one category occupies each rank *j*, divided by the number of movements for which I observed each rank. I then corrected this number by the proportion of individuals in each category. I only retained ranks for which at least five movements were observed. Considering this, the highest analysed rank is 91 for the large group, and 11 for the small group. The homogeneity of the absolute rank distribution was analysed using a Chi square test. The relative frequency distribution per rank and for each category of individuals was analysed using curve estimation tests [66]. Different functions (parabolic, inverse parabolic, linear and cubic) were tested and only the best-fitting function was retained to explain the observed distribution (based on R^2^. R^2 ^are adjusted according to the number of free parameters [67-69]). A linear distribution of ranks indicates that a category has the same probability to occupy all ranks. A parabolic distribution (U-shape) indicates that a category has more probability to be in the front and at the back of the movement. On the contrary, an inverse parabolic distribution (n-shape) suggests that a category is located in the middle of the progression rather than in the front or at the back. I also compared these different distributions between groups using the same test.

Communication: Using the Chi square test, I first analysed if the frequency of vocalisations differed between moving context and stationary context, and whether the emission of vocalisations was followed by an initiation or not. Individuals took 15 minutes to consider a vocalisation linked to a subsequent initiation (in agreement with definitions above). I then assessed whether vocalisations were more frequent during morning departures, in comparison with other movements, using a Mann-Whitney test. I also analysed if vocalisations are emitted more before or after an initiation using a Wilcoxon test. Their frequency was then correlated with the number of followers and the joining time of all participants using a Spearman rank correlation test. I did not test this correlation for joining time in the large group, since group members rarely moved all together, and the number of values with a similar number of followers was too weak to make the statistical testing of this correlation possible. This was not the case for the small group, since the majority of movements concerned the entire group. Lastly I compared the frequency of vocalisations per movement between both groups using a Mann-Whitney test.

The significance level was set at 0.05. All tests were two-tailed. I carried out statistical tests using SPSS 10.00 (SPSS Inc., Chicago, IL, USA). Means are ± SE.

## Results

### Leadership

**Figure 1:**
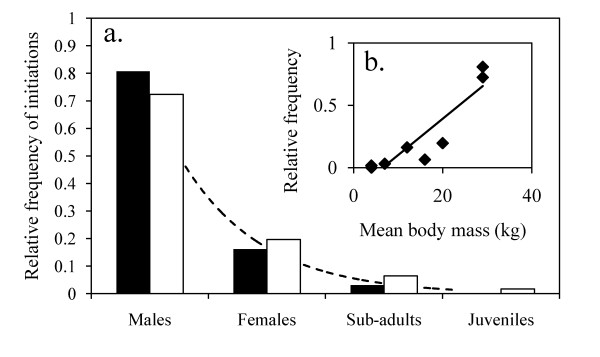
**Leadership in the two groups of baboons **(a) Relative frequency of initiations per category of individuals in the small group (black bars) and in the large group (white bars) of chacma baboons. The dotted line is the leadership distribution according to the category and follows an exponential function. (b) Relative leadership frequency according to the mean body mass of each category of individuals. Lozenges are observed data. The line represents the linear relationship between the two variables.

**Figure 2:**
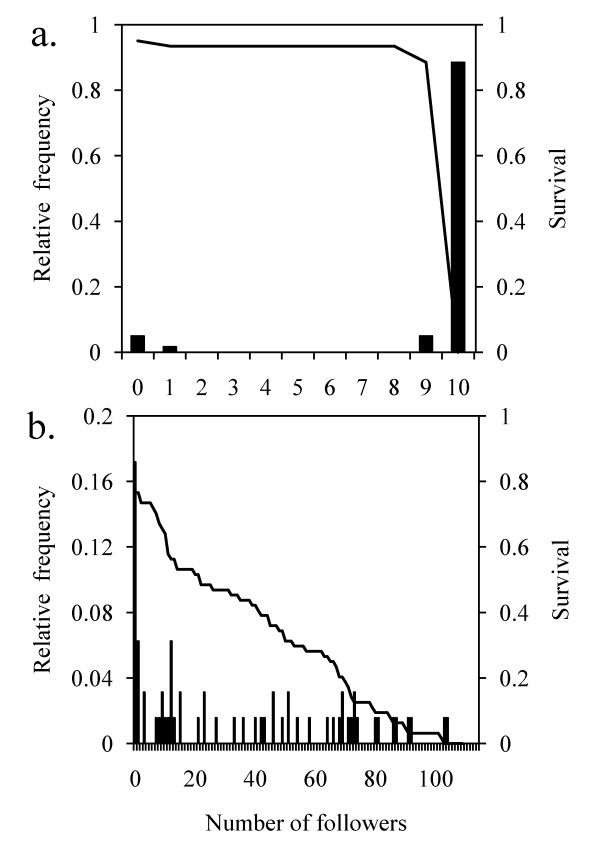
**Frequency of initiations according to the number of followers**. Relative frequency (bars) and survival analysis (line) in (a) the small group and (b) the large group.

Leadership is not equally distributed between age and sex categories in the small group of chacma baboons (χ^2 ^= 362, df = 2, P < 0.00001). The adult male initiated more than adult females, who initiated more than sub-adults and juveniles. Juveniles never initiated movements and sub-adults only did so once. This distribution of leadership (categories are ranked from the highest leadership rate to the slowest one) follows an exponential law (R^2 ^= 0.99, F_1,2 _= 304, P = 0.003, y = 3.225e^-1.482x^, Figure 1a). There is no difference in the number of followers between movements initiated by the adult male and those initiated by adult females (Mann-Whitney test: Z = -0.870, N_male _= 27, N_female _= 32, M_male _= 10.62 ± 0.37, M_female _= 10.06 ± 0.52, P = 0.385). I obtained the same result for the joining time of all participants (Mann-Whitney test: Z = -0.200, N_male _= 27, N_female _= 32, M_male _= 381.67 ± 78.54, M_female _= 374.35 ± 72.10, P = 0.841). Only three failed start attempts were observed (4.9% of cases, two attempts by adult females, one attempt by an adult male) and one movement with one follower (1.6%, in adult females). 88.5% of movements included all group members and 4.9% included ten individuals, showing that the group is cohesive [41,44]. Indeed, a survival analysis showed that the inverse cumulative distribution is non linear and follows a sigmoid function with a threshold equal to the number of group members (R^2 ^= 0.90, F_1,9 _= 70, P = 0.00005, $y=\frac{0.95}{{\left(\frac{x}{11}\right)}^{30}}$, Figure 2a).

In the large group, leadership is not equally distributed between age and sex categories (χ^2 ^= 527, df = 3, P < 0.00001) and follows an exponential law (R^2 ^= 0.99, F_1,2 _= 1706, P = 0.001, y = 2.4874e^-1.245x^, Figure 1a). The adult females with infants initiated fewer movements than females without infants (χ^2 ^= 20, df = 1, P < 0.00001). No difference was observed in the number of followers between the different categories of individuals (K-W test: U = 4.206, N = 64, P = 0.240). As far as the distribution of the number of followers is concerned, all cases were observed, with 11 failed initiations (17.2% of initiations) and 53 initiations (86.8%) attracting 2 to 101 followers. I then categorised the number of followers by intervals of 20 (1-20, 21-40, 41-60, 61-80, 81-105) and found that the frequency of collective movements is the same between each of these categories (K-W test: U = 7.260, N = 53, P = 0.123). Indeed, a survival analysis showed that the inverse cumulative distribution is linear (R^2 ^= 0.95, F_1,103 _= 694, P < 0.00001, y = -0.0071x + 0.6991, Figure 2b). All sub-group sizes during movements were observed at the same frequency.

Both groups have the same leadership distribution (χ^2 ^= 2.3, df = 3, P = 0.551, Figure 1a). The higher the mean body mass of categories, the more they are seen to lead (R^2 ^= 0.83, F_1,6 _= 171, P = 0.002, y = 0.291x - 0.1898, Figure 1b).

### Joining and order during progression

**Figure 3:**
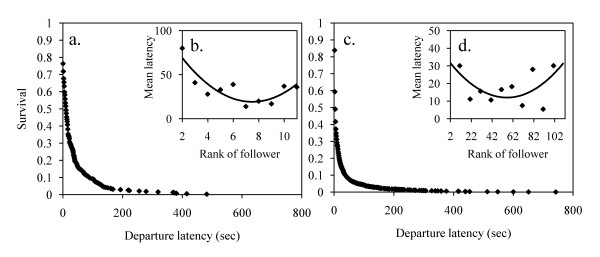
**Distributions of departure latencies**. Survival analysis of departure latencies in (a) the small group and (c) the large group. Mean departure latency according to the rank of progression in (b) the small group and (d) the large group. Lozenges are observed data. The line is the relationship between the two variables and follows a parabolic function.

**Figure 4:**
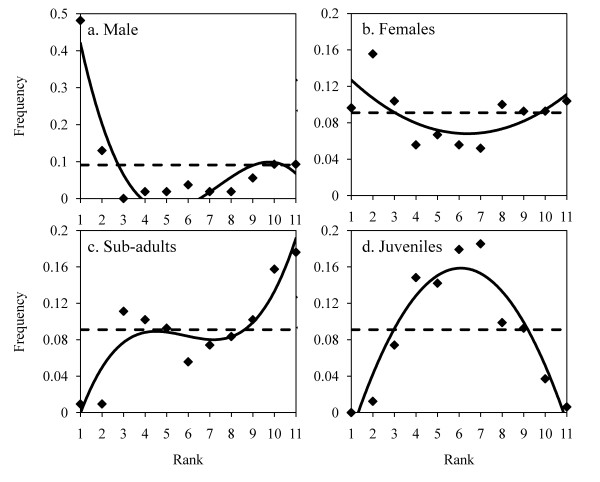
**Relative frequency of observations according to the rank of progression for the small group and per category of individuals**. Lozenges are observed data. The dotted line represents the distribution of frequencies under the hypothesis that there is no specific order (homogeneous distribution). The continuous line represents the observed relationship between the two variables.

Survival analysis in the small group showed that the inverse cumulative distribution of departure latencies of joiners follows an exponential law (R^2 ^= 0.91, F_1,95 _= 908, P < 0.0001, y = 0.3553e^-0.011x^, Figure 3a) better than a linear one (R^2 ^= 0.44, F_1,95 _= 75.58, P < 0.0001, y = -0.0013x + 0.3252), meaning that the probability to join the movement is constant per time unit and equals 0.011 [38,41,45,70]. Departure latency distribution according to the rank of followers follows a parabolic curve (R^2 ^= 0.75, F_1,8 _= 26, P = 0.001, y = 1.6515x^2 ^- 24.761x + 112.04, Figure 3b). I calculated the observation frequencies per rank for each category of individuals. The distribution of absolute frequency according to rank during progression is not homogenous for the adult male (χ^2 ^= 100, df = 9, P < 0.0001), but follows a cubic function (Table 1, Figure 4a). The male was more often located at the front (specifically at the first rank) or at the back of the progression than in the middle. I obtained the same result for adult females. The distribution is not homogeneous (χ^2 ^= 28, df = 10, P = 0.002) but follows a parabolic law (Table 1, Figure 4b). Adult females occupied positions at the front and back of the progression more than in the middle. Sub-adults also had specific ranks: the distribution is not homogenous (χ^2 ^= 33, df = 10, P = 0.0003) but follows a cubic function (Table 1, Figure 4c), meaning that sub-adults were rarely observed at the front of the progression but were observed at the back of the progression in the majority of cases. Lastly, the distribution of juvenile ranks is not homogenous either (χ^2 ^= 64, df = 9, P < 0.0001) and follows an inverse parabolic law (or cubic, Table 1, Figure 4d). Contrary to other group members, juveniles were located in the central part of the progression.

**Table 1:** Curve estimations of the distribution of individual frequency per rank for each age-sex category in the small group of chacma baboons

Category	Function	R^2^	F	P	equation
	Linear	0.19	2.19	0.172	y = -0.0175x + 0.1926
Adult male	Parabolic	0.68	19.71	0.002	y = 0.0104x^2 ^- 0.1429x + 0.4642
	**Cubic**	**0.86**	**54.96**	**< 0.0001**	**y = -0.0025x^3 ^+ 0.0547x^2 ^- 0.3648x + 0.7329**

	Linear	0.03	0.279	0.610	y = -0.0079x + 0.4902
Adult females	**Parabolic**	**0.41**	**141.24**	**< 0.0001**	**y = 0.0101x^2 ^- 0.1294x + 0.7534**
	Cubic	0.41	6.34	0.033	y = 3E-05x^3 ^+ 0.0096x^2 ^- 0.1268x + 0.7503

	Linear	0.60	13.69	0.005	y = 0.0244x + 0.0303
Sub-adults	Parabolic	0.61	14.09	0.005	y = 0.0009x^2 ^+ 0.0133x + 0.0544
	**Cubic**	**0.82**	**39.43**	**0.0001**	**y = 0.0019x^3 ^- 0.034x^2 ^+ 0.1885x - 0.1577**

	Linear	0.01	0.03	0.868	y = 0.0035x + 0.2448
Juveniles	**Parabolic**	**0.88**	**71.02**	**< 0.0001**	**y = -0.021x^2 ^+ 0.255x - 0.3001**
	Cubic	0.88	68.09	< 0.0001	y = 9E-05x^3 ^- 0.0226x^2 ^+ 0.2635x - 0.3103

Results in bold indicate the best-fitting function explaining the distribution. R^2 ^are adjusted according to the number of free parameters.

**Figure 5:**
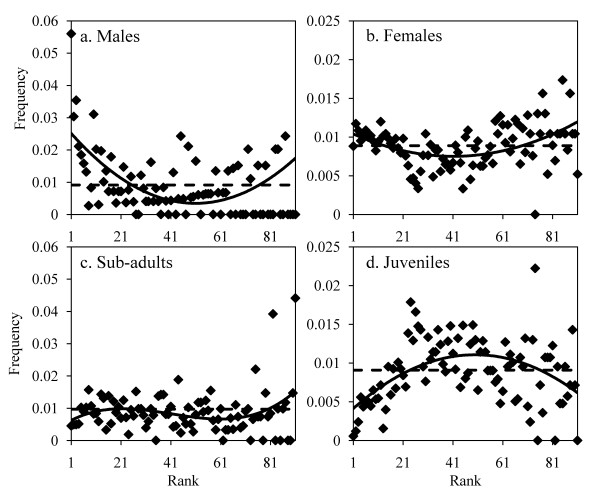
**Relative frequency of observations according to the rank of progression for the large group and per category of individuals**. Lozenges are observed data. The dotted line represents the distribution of frequencies under the hypothesis that there is no specific order (homogeneous distribution). The continuous line represents the observed relationship between the two variables.

The large group seems to be organized in the same way as the small group. Indeed, I observed the same rule underlying the joining and order of group members. The inverse cumulative distribution of departure latencies of joiners follows an exponential law (R^2 ^= 0.90, F_1,153 _= 1269, P < 0.0001, y = 0.1383e^-0.009x^, Figure 3c) better than a linear one (R^2 ^= 0.25, F_1,153 _= 52.28, P < 0.0001 y = -0.0004x + 0.1402): the probability of joining the movement is constant per time unit and equals 0.009. The distribution of departure latencies according to the rank of joiners follows a parabolic curve (R^2 ^= 0.43, F_1,9 _= 6.6, P = 0.030, y = 0.0067x^2 ^- 0.7489x + 33.081, Figure 3d). Adult males were more often observed at the front or back of the progression: the frequency distribution per rank is not homogeneous (χ^2 ^= 309, df = 66, P < 0.00001) and follows a parabolic function (Table 2, Figure 5a). The frequency distribution per rank in adult females is not homogeneous either (χ^2 ^= 512, df = 89, P < 0.00001) and also follows a parabolic function (Table 2, Figure 5b). However, among adult females, the distribution for females without infants is heterogeneous (χ^2 ^= 432, df = 82, P < 0.0001) and follows a parabolic law (R^2 ^= 0.18, F_1,89 _= 22, P < 0.0001, y = 2^E-06^x^2 ^- 0.0001x + 0.0106) whilst the distribution for females with infants is homogeneous (χ^2 ^= 26, df = 63, P = 1.00). Sub-adults occupied certain ranks more than others (χ^2 ^= 137, df = 84, P < 0.0001) but no function fits with the distribution, even the cubic curve (Table 2, Figure 5c). The distribution in juveniles is not homogeneous (χ^2 ^= 304, df = 80, P < 0.0001) and follows an inverse parabolic curve (Table 2, Figure 5d).

**Table 2:** Curve estimations of the distribution of individual frequency per rank for each age-sex category in the large group of chacma baboons

Category	Function	R^2^	F	P	equation
	Linear	0.04	4.42	0.038	y = -3^E-05^x + 0.0119
Adult males	**Parabolic**	**0.19**	**23.43**	**< 0.0001**	**y = 9^E-06^x^2 ^- 0.0009x + 0.026**
	Cubic	0.08	9.35	0.003	y = -2^E-08^x^3 ^+ 1^E-05^x^2 ^- 0.001x + 0.0268

	Linear	0.14	16.17	0.0001	y = 4^E-05^x + 0.0072
Adult females	**Parabolic**	**0.32**	**46.35**	**< 0.0001**	**y = 2^E-06^x^2 ^- 0.0001x + 0.0105**
	Cubic	0.21	28.50	< 0.0001	y = -8^E-09^x^3 ^+ 3^E-06^x^2 ^- 0.0002x + 0.011

	Linear	0.00	0.40	0.529	y = 2^E-05^x + 0.0079
Sub-adults	Parabolic	0.02	1.45	0.231	y = 2^E-06^x^2 ^- 0.0001x + 0.0101
	Cubic	0.03	2.64	0.108	y = 1^E-07^x^3 ^- 1^E-05^x^2 ^+ 0.0004x + 0.0058

	Linear	0.013	0.50	0.479	y = -2E-05x + 0.0093
Juveniles	**Parabolic**	**0.25**	**35.21**	**< 0.0001**	**y = -3^E-06^x^2 ^+ 0.0003x + 0.0038**
	Cubic	0.17	21.67	< 0.0001	y = 9E-08x^3 ^- 2E-05x^2 ^+ 0.0008x - 0.0005

Results in bold indicate the best-fitting function explaining the distribution. R^2 ^are adjusted according to the number of free parameters.

The inverse cumulative distribution of departure latencies in the small group is correlated to that of the large group (R^2 ^= 0.86, F_1,808 _= 5290, P < 0.0001, 1.2222x + 0.0127). The exponential coefficient of these distributions (y = 0.3553e^-0.011x ^for the small group; y = 0.1383e^-0.009x ^for the large group; see above and Figure 3a, c) corresponds to the mean probability to join a movement, and is 0.009 for the members of the small group and 0.011 for members of the large group process. The functions obtained in the four categories of individuals for the small group were then transposed to the large group for the calculation of simulated frequencies per rank for each category. I then compared these simulated frequencies to the frequencies obtained in the large group. Results showed that the distributions are correlated (R^2 ^= 0.68, F_1,354 _= 936, P < 0.0001, y = 0.9706x + 0.0165) showing that members of both groups used similar rules of progression during collective movements.

### Communication

The small group did not display more movements with loud calls (44.2%) than without loud calls (55.8%) (χ^2 ^= 0.5, df = 1, P = 0.484). Similarly, movements with grunts (41.3%) were no more numerous than those without grunts (58.7%) (χ^2 ^= 1.5, df = 1, P = 0.238). However, a movement consistently followed the emission of loud calls (100% of cases), whilst only 87.1% of grunt emissions were followed by a movement (the difference observed with/without grunts is significant: χ^2 ^= 17, df = 1, P < 0.00001). There were more loud calls (Mann-Whitney test: Z = -2.319, N_morning _= 10, N_other _= 51, M_morning _= 5.44 ± 2.47, M_other _= 1.68 ± 0.65, P = 0.038) and more grunts (Mann-Whitney test: Z = -2.952, N_morning _= 10, N_other _= 51, M_morning _= 31.33 ± 21.65, M_other _= 2.84 ± 0.66, P = 0.003) during the morning departures compared to other movements. There were also more loud calls (Wilcoxon test: Z = -3.359, N_before _= N_after _= 61, M_before _= 2.96 ± 0.74, M_after _= 0.15 ± 0.10, P = 0.001) and more grunts (Wilcoxon test: Z = -3.642, N_before _= N_after _= 61, M_before _= 8.51 ± 2.20, M_after _= 0.52 ± 0.31, P = 0.0003) prior to initiations than after initiations. Grunts do not appear to be more numerous prior to loud call emissions than after them (Wilcoxon test: Z = -0.827, N_before _= N_after _= 12, M_before _= 19.58 ± 16.71, M_after _= 4.83 ± 1.62, P = 0.408). The number of loud calls is not correlated to the success of the movement, either in terms of number of followers (Spearman rank correlation: rs = -0.119, N = 61, P = 0.400) or joining time for all participants (Spearman rank correlation: rs = -0.243, N = 57, P = 0.160). The same result is obtained for the relationship between the number of grunts and the number of followers (Spearman rank correlation: rs = 0.005, N = 61, P = 0.972) and for the joining time of all participants (Spearman rank correlation: rs = 0.101, N = 57, P = 0.562).

In the large group, movements with loud calls are no more numerous (56%) than those without loud calls (44%) (χ^2 ^= 0.7, df = 1, P = 0.396). Similarly, the group did not display more movements with grunts (62%) than without grunts (48%) (χ^2 ^= 2.8, df = 1, P = 0.090). Emissions of loud calls are more often followed by a movement (71.4%) than by no movement at all (28.6%) (χ^2 ^= 12.8, df = 1, P = 0.0003). However, emissions of grunts are no more frequently followed by a movement (67.8%) than by no movement at all (32.2%) even if there is a statistical tendency (χ^2 ^= 3.6, df = 1, P = 0.059). The frequency of loud calls is higher when followed by a movement (Mann-Whitney test: Z = -2.331, N_withmovement _= 61, N_withoutmovement _= 20, M_withmovement _= 2.66 ± 0.61, M_withoutmovement _= 1.10 ± 0.57, P = 0.02), whilst there is no difference concerning the frequency of grunts (Mann-Whitney test: Z = -0.309, N_withmovement _= 61, N_withoutmovement _= 20, M_withmovement _= 2.38 ± 0.77, M_withoutmovement _= 5.5 ± 4.16, P = 0.759). There are more loud calls during morning departures than during other movements (Mann-Whitney test: Z = -2.706, N_morning _= 14, N_other _= 50, M_morning _= 4.09 ± 1.48, M_other _= 2.25 ± 0.66, P = 0.007) but not more grunts (Mann-Whitney test: Z = -0.470, N_morning _= 14, N_other _= 50, M_morning _= 1.55 ± 0.91, M_other _= 2.26 ± 0.66, P = 0.962). There are more loud calls (Wilcoxon test: Z = -2.652, N_before _= N_after _= 64, M_before _= 3.32 ± 0.65, M_after _= 1.67 ± 0.67, P = 0.008) and more grunts (Wilcoxon test: Z = -2.019, N_before _= N_after _= 64, M_before _= 2.81 ± 0.68, M_after _= 1.13 ± 0.38, P = 0.044) prior to initiations than after initiations. Grunts are no more numerous prior to the emission of loud calls than after it (Wilcoxon test: Z = -1.686, N_before _= N_after _= 12, M_before _= 4.83 ± 1.67, M_after _= 1.25 ± 0.51, P = 0.092). The number of loud calls (Spearman rank correlation: rs = -0.167, N = 64, P = 0.361) and the number of grunts (Spearman rank correlation: rs = -0.076, N = 64, P = 0.678) are not correlated to the number of followers.

Direct comparison of the two groups in terms of absolute frequencies of loud calls and grunts per movement did not reveal any difference between groups for loud calls (Mann-Whitney test: Z = -0.995, N_small _= 61, N_large _= 64, M_small _= 2.41 ± 0.67, M_large _= 2.66 ± 0.61, P = 0.320) but showed that the small group emitted more grunts per movement than the large group (Mann-Whitney test: Z = -2.345, N_small _= 61, N_large _= 64, M_small _= 4.09 ± 0.87, M_large _= 2.38 ± 0.77, P = 0.019).

## Discussion

This study is the first to simultaneously study leadership, progression order and communication during movements in two different-sized groups of baboons. Whatever the group size, group members seem to show the same rules of decision making. Even if we need to confirm this result in other groups, the latter is very surprising given existing theoretical literature which suggests that small and large groups should display different mechanisms of communication and organization during movements [4,71]. This study showed that in both groups, adult males are more prone to lead the group, even if adult females and sub-adults can also initiate movements. However, there is no difference between individuals of different sex or age as far as the success of initiations is concerned. During progression, individuals seem to join the movement through mimetism or social amplification [37,38,41,45,70]. The parabolic shape of the distribution of latencies is typically the signature of a mimetic process where an individual displayed a behaviour according to the number of individuals already performing it [38,41,72]. If joining a movement followed an independence process (individuals are not influenced by their conspecifics) or a leadership process (the probability to join the movement only depend on the movement initiator), the distribution of latencies would more follow a linear function [40,42]. However, joining order is not random, with adult males and adult females being located more often at the back or front of the movement, whilst juveniles are in the centre of the progression. Lastly, baboons seem to use vocalisations on a context-dependent basis: grunts and loud calls were emitted more often before the initiations of movement than after an initiation or within other contexts. The results are similar in both groups, despite the difference in group size.

Literature often associates leadership, or the frequent initiation of movements, with either dominance of group members or the philopatric sex [3,9,10,45,73]. Few studies have showed the relationship between leadership and nutrient requirements [22,74-76]. In this study, adult males initiated more movements than adult females, sub-adults or juveniles in both of the groups studied. The same result was found in other studies on baboons [9,11]. However, with the exception of juveniles in the small group, all individuals seem to be able to initiate a movement and are followed to the same extent as adult males. Moreover, the exponential distribution of relative leadership obtained in both groups is corrected by the number of individuals per category. If the leadership is not corrected by this ratio, it is therefore approximately the same for adult males and adult females (48% vs. 48% in the small group; 47% vs. 40% in the large group). Adult males and adult females initiated the same number of initiations (at the group level), but as the ratio of adult males is lower than that of adult females, the relative leadership is higher for males (at the individual level, one adult male initiated more often movements than one adult female). This means that the leadership is distributed among all group members even if it is biased amongst adult males. The advantages of leading a group for an individual are that he or she can not only decide on movement direction but also be the first to access resources [11]. Adult males might also be at the front of the progression because they are larger than other group members and therefore play a role in defending the group from predators or competitor groups. Leading the group might therefore be explained by dominance of individuals - high dominance may allow an individual to decide on direction and have access to resources - or could be explained by the needs of individuals - individuals with the highest needs decide which direction will be taken by the group. These two factors - dominance and needs - are often correlated, and authors mainly conclude that leadership is influenced by dominance. However, a theoretical study shows that a biased leadership in favour of adult males could emerge from differences in nutrient requirement between group members [22]. This theoretical study illustrates leadership distribution following the same exponential distribution as that found not only in this current study but also in another study on baboons [9]. Adult males might be more prone to lead because they have higher nutritional needs, or in other words, because other group members as juveniles, have lower nutritional demands. However, this assumption needs to be confirmed by evaluating the independent ability of dominance rank and body mass to predict the probability of initiating a movement across individuals: if body mass significantly predicts the outcome, but dominance rank does not, one could definitively state that the nutrient-need hypothesis outperforms the dominance hypothesis.

Adult males are more often at the front and back of the progression, and particularly occupy the first ranks of the progression. Adult females occupy the same ranks but to a lesser extent. Sub-adults are found more at the back of the group whilst juveniles are in the central part of the progression. These results have already been found in other groups of baboons [29,31,32] and other groups of primates [14,30]. The main ecological reason suggested to explain this specific order is the protection of juveniles against potential predators and other groups of baboons. Authors have often reported that the high cognitive skills of primates, and particularly of baboons, might allow them to adapt their behaviours (here, the order during progression) to the risk level of their environment [29,34,77]. However, this specific order during progression has also been described in ungulates [27,28,78] and social carnivores [79]. In groups of plains zebras, females (especially those lactating) are more often at the front of movements than sub-adults or juveniles [28]. In African buffalos, juveniles and sub-adults do not decide about movements [27]. Females are also at the front of the movements in a herd of cattle [78]. In social carnivores, females are more often observed at the front of the movement, and juveniles are seldom observed at the front in species such as coatis (*Nasua narica*), mongooses, lions (*Panthera leo*), cap hunting dogs (*Lycaon pictus*) or spotted hyenas (*Crocuta crocuta*) [79]. Another explanation is that simple and local rules could lead to the emergence of these complex patterns during movements [36,37,41,45]. Sueur and colleagues [26,41,70] showed that similar patterns of organization can be explained in macaques and lemurs by their use of mimetic rules based on physiological needs, and mimetism based on social relationships between group members. I also found a mimetic process in this study showing that an amplification rule could also be applied in the two studied groups. Both studied groups displayed the same rules for order during progression, irrespective of size. However, the differences between categories for the order of progression are less pronounced in large groups. There is more noise, firstly because of the large group size (the fluctuations and variations of a collective phenomenon increase with the number of individuals implied in the phenomenon [80]), but probably also due to the existence of sub-groups. Indeed, large troops of baboons are often composed of different sub-groups. These sub-groups are of different sizes. Composed of adult males, females and juveniles, they have their own organization [81-83] leading to multiple combinations at the group level.

Vocal communication is not necessary between members of either group for movement decisions [6,9], nor is this acoustic communication necessary for an initiator to be followed by its conspecifics. In this context, the 'visual contact' between individuals appears to be enough for group coordination [4,9,49] and communication would be only local, which is reminiscent of self-organized processes [36,37,41]. However, this study shows that vocalizations, especially loud calls, are emitted more in the context of collective movements. These loud calls are also more frequently emitted before the initiation of a movement than after it. This result is reminiscent of the vocalisations emitted in female gorillas before the silverback initiated a movement [16], but also the group ceremony preceding initiations in social carnivores [79] or the pre-departure processes also described in ungulates or macaques [10,27,52]. However, it is impossible to conclude from this study that the frequency of these loud calls influences decision-making for an initiation or affects its success. Other studies on baboons have also failed to show a direct effect of vocalisations on the recruitment process [9]. Instead of having a direct effect on the joining process, such as recruiting partners, these vocalizations might only be used to signal the motivation to move, and lead in some way to a better coordination of individuals [6,50,55]. Under the global communication hypothesis, I expected the large group to emit more vocalisations than the small one. However, I found that the small group emitted the same number of loud calls per movement and more grunts than the large group. This seems to show that local communication was used more than global communication between individuals within the large group. Global communication might not be so efficient when the group size becomes too large, as Conradt and Roper [71] have already stipulated. Indeed, as the group size increases, the mean distance between two individuals also increases (and the number of visual or acoustic obstacles) and the probability of information transmission decreases. This leads the communication to be more local than global [4]. Another explanation might be that members of the small group emitted more vocalisations because the cohesiveness in this group is stronger and the reasons to remain cohesive are more numerous than in the large one. There is still a lot to be done if we wish to gain a clear understanding of coordination differences between small and large groups, so further studies are necessary in order to assess coordination mechanisms.

## Conclusion

This study provides a clarification of mechanisms underlying the emergence of leadership and the complex patterns of organization during movements in baboons. However, these rules can also be applied in other species, primates or non primates. Physiological differences between individuals, coupled to social amplification based on social relationships, seem to be crucial factors affecting collective decisions. However, further experimental and theoretical studies are necessary before we can disentangle the influences of the different variables affecting the patterns of collective movements.

## Authors' contributions

CS performed the experiments. CS analyzed the data. CS wrote the paper.
